# Real-World Application of Unhealthy Commodity Industries’ Corporate Political Activity Research: Comment on "Corporate Political Activity: Taxonomies and Model of Corporate Influence on Public Policy"

**DOI:** 10.34172/ijhpm.9077

**Published:** 2025-06-08

**Authors:** Francisca Pulido Valente, Hilson Cunha Filho

**Affiliations:** ^1^Public Health Unit of Amadora, Amadora-Sintra Local Health Unit, Lisbon, Portugal.; ^2^Medicine Department, Faculty of Health Sciences, University of Beira Interior, Covilhã, Portugal.

**Keywords:** Commercial Determinants of Health, Taxonomies, Public Health Policy, Alcohol industry, Portugal

## Abstract

Taxonomies are essential tools for structuring evidence in public health, particularly in rapidly evolving fields like the Commercial Determinants of Health (CDoH). Ulucanlar et al addressed an important gap by proposing taxonomies to systematically document and classify corporate political activity (CPA) across unhealthy commodity industries. In this commentary we reflect on the broader relevance of these frameworks for CDoH research and discuss their real-world applicability through a case study of the Portuguese alcohol industry. Drawing from our empirical findings, we highlight both the practical strengths and challenges we encountered, and propose an additional use: employing CPA taxonomies as communication tools to translate complex corporate strategies into accessible narratives for broader public health audiences. Finally, we identify opportunities for refinement, including developing complementary quantitative metrics and the integration of CPA surveillance into routine public health systems.

## 1. Introduction – Relevance of Taxonomies for Measuring Commercial Determinants of Health

 Research on the Commercial Determinants of Health (CDoH) remains predominantly qualitative, drawing heavily on case studies and social sciences perspective.^1^ While these approaches offer critical insights, they can result in fragmented evidence that is difficult to compare across industries, countries and policy contexts. This makes it challenging to build a cohesive body of knowledge or to respond to corporate practices that shape health outcomes.

 Taxonomies provide much-needed structure to this complex and still-emerging field. They help organize and translate diverse forms of evidence into clear conceptual categories – enhancing comparability, facilitating systematic monitoring, and supporting the design of targeted policy responses.^2^ Their utility is particularly relevant in a field like CDoH, which has yet to be fully theorized^3^ and where corporate practices continuously adapt to evolving regulatory and social environments.

 Recent efforts to quantify CDoH impacts – such as the corporate health impact assessment, the corporate permeation index, and the CDoH index, each combining qualitative and quantitative indicators,^4-6^ reflect a growing demand for more systematic and measurable approaches. However, all of these tools rely on robust conceptual foundations—such as taxonomies—to define what exactly is being measured.

 In this commentary, rather than reiterating the theoretical justification for taxonomies, we focus on their real-world application.

## 2. Practical Relevance – Reflections From Applying the Taxonomies

 The taxonomies proposed by Ulucanlar and colleagues’^7^ study represent an important theoretical contribution to the study of corporate political activity (CPA). By categorizing both how corporate actors frame public discourse and how they intervene in policy formulation, these frameworks offer a structured way to document and contest corporate influence. Crucially, they also expose strategic similarities across unhealthy commodities industries, highlighting a recurring playbook that adapts narratives to different products while using common patterns of influence.

 To explore the real-world relevance of these taxonomies, we applied them to the Portuguese alcohol industry – identified in 2019 as an obstacle to implementing cost-effective alcohol control policies in the country.^8^ Our aim was to assess how effectively the CPA taxonomies capture corporate strategies in a concrete policy context, and to reflect on their practical utility for public health actors.

 We conducted a qualitative thematic analysis of publicly available data produced between January 2022 and December 2023, from five dominant alcohol trade associations (wine, beer, spirits). Materials were retrieved from the associations websites (including press releases and reports) as well as from media articles identified through keyword searches (eg, association name + alcohol and/or health). Using a deductive approach, we coded the material according to the CPA *Framing Strategies* taxonomy and extracted illustrative citations. *Action Strategies* were noted when visible, although the data available was limited and did not support systematic analysis.

 In total, 147 statements were analyzed. All categories from Ulucanlar and colleagues’^7^ framing taxonomy were represented.

 The dominant frame—accounting for more than half of the examples—was “Good Actor.” The alcohol industry portrayed itself as a responsible and legitimate stakeholder, a key contributor to economic growth, rural employment, sustainability, and the preservation of cultural heritage and national identity. This strategic positioning served to legitimize its involvement in health policy-making.

 The second most prevalent category was “The Unacceptable, Bad Solution: Whole Population, Statutory,” in which whole-population regulatory measures—such as mandatory warning labels—were depicted as unnecessary, ineffective, or harmful to society. A smaller but significant portion of narratives framed public health advocates as “Bad Actors,” portraying them as extremist, alarmist, or disconnected from societal realities. Alcohol-related harms were frequently downplayed, with blame shifted onto a minority of irresponsible consumers, and broader structural causes depoliticized. A tokenistic emphasis on promoting “responsible drinking” campaigns reinforced individual behavior changes while opposing statutory regulation.

 Far from being isolated instances of discourse tactics, these findings reflect a consistent effort to shape the alcohol policy landscape in Portugal. The persistence of these narratives since 2019 suggests not only the adaptability of CPA, but its deep institutional embedding in national public discourse. This aligns with international evidence that unhealthy commodity industries engage in long-term discursive strategies to normalize their influence and pre-empt regulation.

 Importantly, beyond their analytical value, our experience also suggested an underexplored function of CPA taxonomies: their use as communication tools. By organizing industry narratives into simplified, recognizable frames—such as “Good Actor” vs. “Bad Actor,”—these taxonomies helped translate complex CPA strategies into structured narratives that were accessible even to public health professionals unfamiliar with CDoH. This communicative function enhanced engagement, facilitated clearer discussions about corporate influence and supported awareness-raising. Crucially, it enabled us not only to analyze corporate strategies but also to name, illustrate, and share them in ways that could inform public health action.

## 3. Challenges in the Real-World Application of CPA Taxonomies

###  Blended Narratives and Overlapping *Framings Strategies*

 One of the main challenges we encountered was the difficulty of classifying corporate narratives into discrete, non-overlapping categories. As anticipated by Ulucanlar et al, CPA strategies are inherently dynamic, evolving to resist regulation, maintain legitimacy, and respond to shifting public health norms. This hyper-adaptability is, as the authors noted, both a strategic asset for corporations and a potential vulnerability when exposed.

 In our data set, industry messages often blended multiple frames in the same statement. For example, claims promoting self-regulation as a sign of responsibility (“Good Actor”) were frequently accompanied by arguments dismissing statutory regulation as ineffective (“Bad Solution”) and by portrayals of public health advocates as extreme or unreasonable (“Bad Actor”). One illustrative example was: *“We believe self-regulation, along with public awareness campaigns, are the best way to promote responsible consumption without hurting businesses.”*

 These hybrid narratives are not accidental – they are crafted to maximize persuasive reach across multiple spheres. This overlap complicates both the analytical application of the taxonomies and the development of effective counterstrategies. While taxonomies help simplify and clarify corporate strategies, their rigid use may obscure the complex, relational nature of real-world CPA. Corporate messaging is often hybrid, ambiguous, and mutually reinforcing—designed not only to influence specific policies but to shape the broader political discourse in which those policies are debated.

 Although taxonomies make corporate influence more visible, their simplification can also flatten strategic complexity. Ulucanlar et al acknowledged these limitations, yet their current framework may still lack the nuance necessary to fully capture how overlapping corporate tactics interact and enhance one another. Moving forward, CPA analysis should go beyond discrete categories and incorporate more context-sensitive approaches that reflect how corporate strategies operate in combination.

###  Limited Visibility of *Action Strategies*

 Unlike *Framing Strategies* – which are often visible in public statements, social media, and advertising, *Action Strategies*— such as regulatory capture, venue shifting or revolving door practices—are more covert and difficult to trace. In our study of the Portuguese alcohol industry, reliance on publicly available sources limited our ability to systematically detect these forms of influence. Certain forms of CPA, like financial ties between corporations and policy-makers, the use of third-party allies, and funding of scientific research may be concealed from public view and require in-depth investigation to uncover. Researchers must rely on leaked communications, financial disclosures, and whistleblower accounts, which might be incomplete or unavailable. Moreover, strategies such as regulatory preemption—where stronger local laws are overridden—or venue shifting—where decision-making is moved to more industry-friendly settings—tend to unfold in subtle ways, beyond the reach of standard public health monitoring.

 These challenges highlight that the utility of the CPA taxonomies depend not only on analytical clarity but also on data access. The less visible the strategy, the harder it is to capture within a structured framework. This calls for investigative tools capable of mapping CPA that extends beyond formal public discourse.

###  Third-Party Actors and Structural Alignment

 A third challenge lies in how the taxonomies treat third-party actors. While Ulucanlar et al acknowledge the use of intermediaries—such as front groups, academics, industry-funded non-governmental organizations—these are primarily conceptualized as direct extensions of corporate strategy. In practice, however, such actors may operate with a degree of autonomy, reproducing corporate framings without explicit coordination.

 For instance, a minister opposing alcohol pricing and taxation policies may echo industry narratives not because of explicit collaboration, but due to ideological alignment, shared economic interests, or institutional inertia. These forms of structural alignment are difficult to trace yet can be highly influential. Over time, such actors can acquire their own institutional power, reinforcing industry framings even in the absence of direct and active corporate involvement.^9^ The taxonomies do not clearly distinguish between corporate-led and institutionally driven influence. Expanding them to capture broader networks of aligned power—including political and institutional actors who defend or amplify industry positions—would increase their relevance for public health surveillance and strategic response.

 This limitation is particularly relevant in public health, where corporate power has not traditionally been a central focus. As a result, the scope, depth, and persistence of corporate influence in health policy remain underestimated and poorly understood.^10^

## Conclusion and Call to Action

 The CPA taxonomies developed by Ulucanlar et al make an important contribution to public health research and practice. They provide structured tools for identifying, categorizing, and communicating CPA—a critical step in making CDoH more visible, comparable, and actionable. Our experience applying these taxonomies to the Portuguese alcohol industry underscores their dual function: they are not only analytical instruments but also powerful communication tools capable of translating complex corporate strategies into accessible narratives for broader public health audiences.

 However, CPA in the real world is dynamic, hybrid, and strategically adaptive. While the taxonomies offer a solid foundation, they do not capture the full complexity, intensity, or evolution of CPA over time.

 To address these limitations, future efforts should focus on developing complementary quantitative tools – such as CPA scoring systems or indices aligning with the taxonomies- that allow for the measurement of intensity, prevalence, and trends.

 For CPA taxonomies to be used routinely and autonomously in public health practice, investment is also needed in capacity building, digital infrastructure, and the integration of CPA surveillance into broader institutional frameworks. Open-access platforms with real-time feedback loops, supported by automated tools, could make taxonomies more adaptive. These systems could function similarly to vector control surveillance in epidemiology–enabling near real-time tracking of CPA strategies and corporate influence across contexts.

 Ultimately, CPA is not random; it is structured, systematic, and context-responsive. Recognizing this should shift public health away from passive documentation toward institutionalized CPA surveillance—building intelligent, proactive systems capable of detecting, and analyzing corporate interference, much like tracking an evolving epidemic.

 Addressing the CDoH also requires sustained interdisciplinary and intersectoral collaboration. This includes embedding CPA analysis into policy-making processes, research agendas, and public health education. Importantly, using any form of CPA monitoring remains particularly challenging in places where awareness and dialogue about CDoH are still limited. As others have noted, building capacity through the education and training of the public health workforce is essential.^11^

 Without coordinated and institutionalized action, the CDoH will continue to undermine public health outcomes. Building resilient, transparent systems capable of resisting corporate interference must become a core function of 21st-century public health.

## Acknowledgments

 We thank the anonymous reviewers for their insightful and constructive comments.

## Ethical issues

 Not applicable.

## Conflicts of interest

 Authors declare that they have no conflicts of interest.

## Disclaimer

 The views expressed in this manuscript are those of the authors and do not necessarily reflect the official policy or position of any affiliated institutions.
